# Neighbourhood cohesion and chronic loneliness in the UK: analysis of the UK Household Longitudinal Study

**DOI:** 10.1093/geroni/igaf122.3978

**Published:** 2025-12-31

**Authors:** Christina Victor, Isla Rippon

**Affiliations:** Brunel University London, Guildford, England, United Kingdom; Brunel University London, Guildford, England, United Kingdom

## Abstract

Loneliness has been identified as a major global public health challenge. There is substantial evidence reporting prevalence and risk factors for loneliness in older adults. Longitudinal evidence about loneliness is less well established and there is no consensus on the typology characterising longitudinal loneliness trajectories. Our evidence examining neighbourhood level factors is limited mostly focussing upon walkability or access to blue-green spaces. We investigate the role of neighbourhood cohesion as a ‘protective’ factor against experiencing chronic loneliness and focuses upon two under-researched groups of older adults, minoritised ethnic groups and sexual minorities. We use data for 10,916 adults aged 50+ who participated in waves 9-11- and 13 of the United Kingdom Household Longitudinal Survey (UKHLS). Loneliness was measured using the three-item UCLA scale with a score of 6+ defining loneliness and neighbourhood cohesion by the Buckner scale. We define four loneliness trajectories: not lonely, transient (lonely once), fluctuating (two or three occasions of loneliness) and chronic (lonely at every wave). Chronic loneliness is higher among black, Indian, white Irish and mixed ethnicity populations compared to white British respondents. Sexual minority participants had higher levels of chronic loneliness than heterosexual participants. Neighbourhood cohesion is highest for the white Irish group and heterosexual groups and lowest for mixed race and sexual minority participants. High neighbourhood cohesion is protective against chronic loneliness for white British, Irish and for both sexual minority and majority groups. Adjustment age, sex, living alone, material resources and mental well being attenuated these relationships.

